# Supplementary material for: Are carbon emissions trading and green financial instruments synergistic? -Comprehensive quantitative research based on content analysis
Source: PLoS One. 2024 Mar 7;19(3):e0298601. doi: 10.1371/journal.pone.0298601 (PMC10919601; doi:10.1371/journal.pone.0298601)
Supplement: S1 Data — (ZIP) [file pone.0298601.s001.zip › Data/地级市Z_F工作报告.zip!/╝¬┴╓/╦─╞╜╩╨/└┤╫╘╣1⁄2╓┌║┼í╛┬φ┐╦╩2╛▌═°í┐_2008.docx]

各位代表：

　　现在，我代表市政府，向四平市第六届人民代表大会第一次会议报告工作，请予审议。

一、过去四年工作回顾

　　过去四年，在省委、省政府和市委的正确领导下，在人大、政协的监督支持下，经过全市人民的共同努力，取得了经济社会发展新成就。

　　——国民经济快速健康发展。预计今年全市生产总值完成471亿元，年均增长17.1%。三次产业的比例由2003年的40.6：28.4：31调整为34：35：31。一般预算全口径财政收入28.2亿元，年均增长14.9%；市区一般预算全口径财政收入15.1亿元，年均增长20.1%。工业经济运行质量明显提高，规模以上工业企业完成增加值120亿元，年均增长24.5%；利润8.5亿元，年均增长110%。农村经济全面发展，粮食产量连续四年超越110亿斤阶段性水平。服务业不断发展壮大。金融对经济支持力度加大，预计全市贷款余额达到302亿元。节能降耗取得成效，主要污染物排放得到有效控制。

　　——大上项目、全民招商成效显著。预计今年全社会固定资产投资达到220亿元，四年累计完成固定资产投资507亿元，年均增长41.2%。红嘴集团、四平烟厂、双辽发电厂、四平热电厂、公主岭黄龙、四平昊华、双辽福耀玻璃、伊通金圆水泥等较大企业发展步伐加快；昊华二期、天成玉米二期、新加坡生物科技园等大项目相继开工建设；双辽电厂二期和四平热电厂二期项目明年一季度可破土动工，这些企业和项目对经济增长起到积极拉动作用。四年来，全市共引进1000万元以上项目980个，其中亿元以上项目150个。争取国债项目105个，国债资金8.55亿元。争取振兴老工业基地项目24个，累计完成投资25.6亿元。预计今年招商引资到位资金150亿元，四年累计招商引资到位资金388.8亿元，年均增长36.2%。

　　——改革开放取得新突破。全市基本完成国企改革任务，厂办大集体改革已经启动。农村综合配套改革、粮食流通体制改革等取得重大进展。民营经济发展迅速，预计到年末全市民营经济可实现主营业务收入410亿元。对外开放进一步扩大，四年累计进出口总额3.16亿美元，年均增长43%。对外经贸交流取得良好成效，开发区带动作用明显增强。

　　——各项社会事业全面进步。教育发展水平进一步提高。科技进步与创新步伐加快。文化事业更加繁荣。城乡医疗卫生服务体系建设、疾病预防控制得到加强。在全国率先推行生育关怀工作。群众性体育活动广泛开展。民政社会福利事业得到发展。广播电视、旅游、人防、普法、地方志、档案、老龄、民族、宗教、防震、知识产权、信息产业和残疾人事业取得新成绩。

　　——城乡基础设施不断完善。四年来，市区共新建和改造12条主干路和近百条支次干路，新建和扩建了10座桥梁；棚户区改造进展顺利，共完成拆迁面积102.01万平方米，回迁楼竣工面积66.25万平方米，建立了城市廉租住房制度；创建国家园林城市取得阶段性成果，人居环境进一步优化；污水处理厂达标运营；四平战役纪念馆新馆建成对外开放。经多方努力，总投资15.4亿元的国道102线四平至长春段一级路和总投资8.2亿元的四平环城一级路建成通车。全市四年累计新建公路3753公里，其中修建村路3047公里，新增通油路（水泥路）行政村495个，通村率76.8%。

　　——人民生活继续改善。认真解决民生问题，预计今年城镇居民人均可支配收入达到12000元，年均增长15.7%；农民人均纯收入达到4100元，年均增长12.6%。四年共开发就业岗位23.87万个，平均每年输出城乡劳动力50万人次。社会保障体系框架初步建立，为10.3万名参加社保离退休职工按时足额发放了养老金，城市低保对象实现了动态管理下的应保尽保，建立并启动了城市居民医疗保险，失业保险进一步完善，城镇登记失业率控制在4.5%以内。二龙山水库综合治理取得成效，水质明显好转。实现市区10万户居民24小时供水。

　　——精神文明和民主法制建设取得新成果。思想道德建设有所提高，城乡精神文明创建活动不断深入。政府自身建设进一步加强。自觉接受人大法律监督、工作监督和政协民主监督。四年共办理人大代表建议444件，政协委员提案686件，办复率100%。加强政府法制工作，坚持依法行政。认真办理来信来访，努力化解各种社会矛盾。国防后备力量建设和双拥共建进一步加强，军政军民关系更加密切。

　　各位代表，四年来全市经济社会保持了良好的发展势头，这是全市上下团结奋斗，共同努力的结果。在此，我谨代表市政府，向全市各族人民、人大代表、政协委员、离退休老同志、各民主党派、各人民团体、工商联、无党派人士、驻平中省直各单位、驻平部队、武警官兵、政法干警和所有关心支持四平发展的各界朋友，表示衷心地感谢和崇高的敬意！

　　回顾过去的四年，我们也清醒地认识到存在的矛盾和问题：一是虽然各项主要经济指标增幅较高，但经济总量还不大，经济质量还不高，特别是财力增长远远抵不住刚性支出的增长，财政仍然比较困难。二是由于财力有限，社会保障压力加大，城市建设欠账较多，在教育、医疗保险、就业、住房、环保、基础设施、公用事业等方面，有很多该办的事情没有能力办。三是农业仍然没有摆脱靠天吃饭的局面，建设现代农业和推进新农村建设需付出艰苦努力。四是城乡低收入群众生活困难，企业改制、征地拆迁等引发的矛盾交织在一起，还存在很多不稳定因素。五是政府自身建设有待进一步加强，干部作风需要进一步转变。对此，我们一定高度重视，采取有效措施，认真加以解决。

　　二、今后五年工作安排

　　今后五年，是完成“十一五”规划和全面建成小康社会的关键时期。全市经济和社会发展的总体思路是：以邓小平理论、“三个代表”重要思想和科学发展观为指导，深入贯彻落实党的十七大精神，以加快发展为主题，以改革开放为动力，以“大上项目、全民招商”为突破口，继续实施工业立市、科教兴市和可持续发展战略，不断优化发展环境，大力发展第三产业，搞好社会主义新农村建设，着力解决民生问题，全力推进四平大发展、快发展，为早日实现四平振兴、人民富裕而努力奋斗。

　　按照市第五次党代会描绘的宏伟蓝图，到2012年全市经济社会发展预期目标是：生产总值年均增长20%以上；人均生产总值达到35000元以上；三次产业结构调整到21：42：37。一般预算全口径财政收入年均增长17%以上；全社会固定资产投资年均增长16%以上；规模以上工业增加值年均增长25%以上；社会消费品零售总额年均增长18%以上；进出口总额年均增长20%以上；城镇居民人均可支配收入和农民人均纯收入年均分别增长12%和13.7%以上；居民消费价格指数每年控制在103%以内；城镇登记失业率保持在4%左右；人口自然增长率控制在2‰以内；万元GDP综合能耗年均下降6%。

　　各位代表，今后五年经济社会发展的任务十分繁重。我们必须解放思想，与时俱进，紧紧扭住经济建设这个中心，聚精会神搞建设，一心一意谋发展。在工作中要紧紧把握以下几个方面：

　　——坚持大上项目，全民招商。把项目建设放在更加突出位置，谋划建设一批投资额度大，带动作用强，发展后劲足，关系经济发展全局的大项目。加强项目管理，搞好科学论证，避免低水平重复建设和无效投入。提高招商引资规模和质量，拓宽对外开放和经济技术合作，扩大利用域外资金和民间投资规模，实现投资主体多元化。

　　——发挥现有优势，提高工业整体发展水平。把工业作为经济工作重中之重，围绕资源优势，着力构建农副产品深加工、建材等资源优势产业；围绕产业优势，做大做强食品、机械、化工、能源、冶金、纺织、医药、高新技术等一批重点企业；围绕产品优势，把玉米淀粉、无水酒精、换热器、汽车配件改装、浮法玻璃、离子膜烧碱、聚氯乙烯、金士百啤酒、宏宝莱饮品等打造成知名品牌和驰名商标。在现有基础上，五年内实现利税超5亿元的企业3户以上，利税超3亿元的企业5户以上，利税超亿元的企业10户以上。

　　——大力发展现代农业，推进新农村建设。加强农业基础设施建设，不断提高粮食生产能力。发展牧业大产业，建设牧业强市。培育一批竞争力、带动力强的农副产品深加工龙头企业，壮大县域经济。提高农民素质，积极推进农村劳动力转移就业。发展农村公益事业，稳步推进社会主义新农村建设。

　　——积极扶持第三产业，促进民营经济腾飞。加大政策引导和扶持力度，加快第三产业发展步伐。进一步完善适合民营企业发展的优惠政策，打造优良环境，促进民营经济加快发展。

　　——实施科教兴市战略，改善经济发展质量。加大教育工作力度，全面提高教育质量和办学水平。推进科技创新，加大科技投入，加快科技体制改革，加速科技成果向现实生产力转化，不断提高科技进步对经济增长的贡献率。

　　——加强能源资源节约和生态环境保护，走可持续发展道路。努力建设节约型社会。加大污染源治理力度，把好新上项目环评关。强化对土地、矿产、森林、水源等自然资源的保护，做到经济发展与改善生态环境相互促进、良性循环。

　　——不断改善民生，让群众共享发展成果。拓宽就业渠道，扩大就业总量。增加城乡居民特别是低收入者的收入，逐步解决城乡低收入家庭住房困难。健全社会保险、社会救助、社会福利、慈善事业相衔接的覆盖城乡居民的社会保障体系。进一步加强人口和计划生育工作。推进医疗卫生体制改革，完善公共卫生和城市社区医疗卫生服务体系，完善新型农村合作医疗制度。加快发展文化、体育等各项社会事业，全面提高城乡居民生活质量。

　　——努力化解矛盾，维护社会和谐稳定。加强思想道德、社会诚信体系建设，促进社会更加和谐。全面开展社会主义法治理念教育，加强社会治安综合治理，依法严厉打击各种犯罪，搞好矛盾纠纷排查调处，防止发生安全生产事故。

　　明年是全面贯彻落实党的十七大精神的第一年，是实施“十一五”规划的关键一年。做好明年工作至关重要。2008年全市国民经济和社会发展的主要预期目标是：生产总值565亿元，增长20%；一般预算全口径财政收入33.2亿元，增长18%；市区一般预算全口径财政收入18.1亿元，增长20%；全社会固定资产投资280亿元，增长27.3%；规模以上工业增加值158亿元，增长32%；社会消费品零售总额181亿元，增长18%；进出口总额1.1亿美元，增长20%；城镇居民人均可支配收入13800元，增长15%；农民人均纯收入4700元，增长14.5%；居民消费价格指数控制在103%以内；城镇登记失业率控制在4.5%以内。人口自然增长率控制在5‰以内。万元GDP综合能耗下降6%。

　　实现上述目标，重点抓好七个方面工作：

　　（一）千方百计增投资上项目。以工业项目为主，集全市之力开发建设一批大项目。加快昊华二期、天成玉米二期、新加坡三达生物科技园、梨树红嘴集团畜产品加工园、精细化工与石药合作项目、伊通石油勘探开发等大项目的建设速度，尽早投产达效。做好双辽发电厂二期、四平热电厂二期、通钢冷轧板二期、公主岭国能生物发电等大项目协调服务，尽早开工建设。继续加强招商引资。创新招商方式，讲求招商质量和效益，降低招商成本，提高招商成功率。吸引大企业大集团来平投资，重点引进对财政贡献大、有后劲的大项目。全年完成招商引资任务180亿元。充分发挥开发区（工业集中区）集聚辐射功能。加快基础设施建设，围绕优势产业引进项目，真正把开发区（工业集中区）建成招商引资平台和工业项目园区，培育新的经济增长极。落实优惠政策，提供优质服务，为企业家和投资者创造更加宽松的软环境。

　　（二）集中力量抓好工业经济。大力支持红嘴集团、四平烟厂、双辽发电厂、四平热电厂、黄龙、昊华、天成等企业加快发展，重点培育一批利税5000万元以上的大企业，加快工业特色园区建设，积极谋划企业上市。鼓励企业自主创新，走产学研结合发展道路，提高装备技术和工艺水平。深入开展“质量兴市”活动，大力培育和发展名牌产品。落实节能减排工作责任制，提高能源资源利用效率。积极扶持和促进中小企业成长，下力量对换热器、汽车配件改装进行整合，使其尽快做大做强。继续深化企业改革，推进厂办大集体改革试点。加强工业经济运行的综合协调，搞好生产要素供需衔接。构建银企对接平台。搞好企业家队伍建设。

　　（三）扎实推进社会主义新农村建设。认真落实粮食生产各项扶持政策，强化耕地保护，提高农业综合生产能力，确保粮食生产稳定增长。搞好农副产品深加工，推进产业化经营，加快发展县域经济。积极培育农村新型合作经济组织，提高农民市场化组织程度。坚持总量扩张与规模化养殖并重，搞好牧业小区建设，强化动物疫病防治，促进畜牧产业健康发展。加强组织，搞好培训，提高技能，提升农村劳动力转移就业水平。积极发展农村二、三产业和多种经营，多渠道增加农民收入。切实搞好中国农业银行面向“三农”金融服务试点工作。加快农村水、电、气、路等基础设施和教育、卫生、文化等公益事业建设。修建村路900公里以上，村村通油路（水泥路）率达到82.4%。

　　（四）大力发展第三产业和民营经济。积极培育和扶持第三产业发展。继续发展商贸、物流和生活服务等传统产业；积极发展旅游、信息、咨询、技术服务、法律服务和中介服务等新兴产业；规范发展金融、保险和房地产业。进一步完善政策，加强服务体系建设，支持民间资本进入所有开放的行业和领域创办实业。引导投资者和社会资本参加企业改制。支持机关事业单位人员领办企业，鼓励在外人员返平创业。规范民营企业依法经营，照章纳税。强化舆论引导，创新择业理念，营造民营经济腾飞的浓厚氛围。

　　（五）加快中心城市建设步伐。搞好新一轮城市总体规划修编，强化城市管理，巩固国家卫生城市成果，提高中心城市辐射功能，带动县城和重点城镇建设。本着量力而行、尽力而为的原则，市区建设主要抓好六件事：一是继续搞好棚户区改造，全面实施廉租住房制度。二是新建东山大街、铁东南一纬路、市府路、解放路、兴平街5条街路，翻建维修天桥街、铁东一经街等10条街路。三是着手建设体育场。四是开工建设生活垃圾处理场。五是平东公铁立交桥建成通车。六是完善城市园林设施，增加人均公共绿地面积，早日实现创建国家园林城市目标。

　　（六）积极发展各项社会事业。调整教育布局，加强教师队伍建设，有效控制中小学生流失，提高教育教学质量。积极支持吉林师范大学提高办学水平，加强校地、校企合作。搞好关键技术和高新技术的开发应用，抓好40个高新技术产业化项目的实施。建立健全人才选拔和引进激励机制，加强人才队伍建设。推进医疗卫生体制改革，提高公共卫生体系建设水平，加快城市标准化社区卫生服务中心建设，完善农村三级卫生服务网络。深入开展生育关怀活动，稳定低生育水平，提高出生人口素质，有效控制出生人口性别比。推进文化事业繁荣发展，抓好文化基础设施建设，发展具有四平特色的文化产业。开展全民健身运动，提高竞技体育水平。积极推进有线电视数字化和广播电视“村村通”工程。依法合理使用土地，保证重点项目用地。坚持开源与节流并重，加强税收征管。牢固树立“过紧日子”思想，把有限资金用在刀刃上。强化审计监督，确保国有资产安全。充分发挥金融机构融资主渠道作用，建立良好的银政银企关系。做好“双拥”工作，巩固双拥模范城成果。认真做好民族、宗教、残疾人事业、普法、外事外联、侨务、对台、统计、气象、防震、知识产权、人防、老龄、档案、地方志等工作。

　　（七）着力解决民生问题。全面贯彻执行《就业促进法》，落实各项扶持政策，深入开展零就业家庭就业援助，全年新开发就业岗位6万个，其中：公益性岗位5000个，安置5.5万人就业。加快完善社会保障体系。全面建立和完善城镇居民基本医疗保险制度，完善新型农村合作医疗制度，切实解决群众看病时遇到的困难和问题。继续扩大养老保险和失业保险覆盖面，依法强制征缴，确保养老金和失业救济金按时足额发放。加大各级财政投入，建立与当地经济发展相适应的城市低保标准和保障资金自然增长机制，实现城乡居民最低生活保障全覆盖。健全贫困学生资助制度，保证困难家庭子女顺利完成学业。切实加强价格监管和调控，保持物价总体水平基本稳定。继续解决好农村饮水安全、泥草房改建等问题。加强环境保护。进一步抓好辽河流域水污染防治，做好二龙山水库等水源地的综合治理，加大对不法排污企业的监管力度，落实污染物总量减排措施，完成各项环保任务指标。狠抓安全生产，预防和减少各类事故发生。积极贯彻实施《劳动合同法》，维护劳动者合法权益。加强信访工作，落实信访责任制，排查化解矛盾纠纷，妥善处置群体性事件。落实社会治安综合治理各项措施，保持严打态势，维护治安稳定，增强群众安全感。加大对传销、制假售假打击力度，维护市场经济秩序。认真贯彻《未成年人保护法》，保护青少年身心健康。强化对餐饮卫生、食品、药品的监管，让广大群众吃上放心食品，用上放心药品。

　　三、切实加强政府自身建设

　　适应新形势新任务的要求，努力建设亲民、高效、诚信、廉洁、法治的服务型政府。

　　心系百姓。牢记全心全意为人民服务宗旨，把群众满意不满意作为政府一切工作的根本出发点和落脚点，设身处地为群众着想，更加关注民生、更加关注弱势群体，多做雪中送炭的事情，切实解决好群众最关心、最直接、最现实的利益问题。

　　真抓实干。学习红嘴精神，增强事业心责任感，求实务实抓实，力戒形式主义，不做表面文章，多干打基础、利长远的事情。深入实际，破解难题，加快节奏，提高效率。对重点工作持之以恒，一抓到底，加大督查督办力度，不达目的不罢休。确保政令畅通，不准推诿扯皮、各自为政，努力提高政府执行力和公信力。

　　依法行政。严格按照法定权限和程序行使行政权力，依法管理经济社会事务。实行政务公开，公平公正，阳光作业，规范操作。健全政府职责体系，推行行政问责制。自觉接受人大法律监督、工作监督和政协民主监督，接受社会公众和新闻舆论监督。认真办理人大代表建议和政协委员提案。凡属重大决策要充分论证和听取各方面意见，坚持科学决策、民主决策。

　　廉洁从政。构建教育、制度、监督并重的惩治和预防腐败体系，从严治政，努力为人民掌好权、用好权。严肃查处各类违法违纪案件，深入开展治理商业贿赂工作。艰苦奋斗，勤俭办事，降低行政成本。切实解决损害群众利益的突出问题，纠正部门和行业不正之风。

　　和谐共进。加强各级政府机关领导班子建设，坚持团结干事，和谐共事，按章办事。教育干部讲学习、讲正气、讲奉献，营造风清气正的良好局面。加强同各民主党派、群众团体、无党派人士的联系，团结一切可以团结的力量，调动一切积极因素，建设和谐四平。

　　各位代表，四平正处在大发展、快发展的关键时期，让我们在市委的正确领导下，在人大、政协的监督支持下，凝聚全市人民的智慧和力量，开拓进取，真抓实干，为早日实现四平振兴、人民富裕而努力奋斗！
